# Cervical Cancer Prevention Among Rural Women in Gujarat, India: A Mixed Methods Study on Risk Factors and KAP (Knowledge, Attitude and Practice)

**DOI:** 10.7759/cureus.69169

**Published:** 2024-09-11

**Authors:** Rohankumar Gandhi, Abhishek Patel, Monika Patel, Sakshi A Sojitra, Tanmay S Kundal, Yogesh Murugan

**Affiliations:** 1 Community and Family Medicine, Shri Meghaji Pethraj (MP) Shah Medical College, Jamnagar, IND; 2 Internal Medicine, Gujarat Medical Education and Research Society (GMERS) Medical College, Valsad, IND; 3 Community and Family Medicine, Gujarat Medical Education and Research Society (GMERS) Medical College, Morbi, IND; 4 Internal Medicine, Shri Meghaji Pethraj (MP) Shah Medical College, Jamnagar, IND; 5 Family Medicine, Gurugobind Government Hospital, Jamnagar, IND

**Keywords:** cervical cancer, india, mixed methods, rural women, screening, sociocultural factors

## Abstract

Background: Cervical cancer causes significant morbidity and mortality among women in India. Despite national screening guidelines, uptake of these tools remains poor, especially in rural areas where complex sociocultural factors are at play. Integrated mixed-methods designs can provide better insights into multilevel barriers influencing screening behaviours. This study aimed to assess the knowledge, attitudes, practices, and sociocultural factors associated with cervical cancer prevention among marginalized rural women.

Methods: A mixed-method study of 400 women aged older than 18 years was conducted using a pretested questionnaire on cervical cancer knowledge, attitudes, self-reported screening practices, and sociodemographic variables. An exploratory qualitative study also interviewed 30 women to explore perspectives on screening using semi-structured guides. The survey data were analyzed via logistic regression, and thematic analysis was performed for the qualitative data. The results were triangulated to enable nuanced interpretation.

Results: Only 27% (108/400) of participants had heard of cervical cancer, and 61% (244/400) were illiterate. Poor knowledge was observed in 83% (332/400) of participants, predicted by early marriage, high parity, low education, and socioeconomic status. Despite 64% (254/400) expressing positive attitudes, only 9% (36/400) reported undergoing cervical cancer screening. None of the participants were vaccinated against human papillomaviruses (HPV). Stigma, gender roles, spousal communication gaps, and access barriers emerged as key qualitative themes. The integrated results highlighted the intersections between villagers’ worldviews and sociocultural norms and between access issues and prevention.

Conclusions: Multifaceted sociocultural challenges underpin the cervical cancer prevention gap among marginalized rural women. Grassroots educational efforts respectfully addressing fears and stigma, along with increased male engagement, community health worker training, and integrated screening services, can promote informed screening decisions among underserved groups.

## Introduction

Cervical cancer is a major public health problem worldwide, especially in low- and middle-income countries (LMIC) [1]. It is the fourth most common cancer among women worldwide, with an estimated 604,000 new cases and 342,000 deaths in 2020 [2]. In India, cervical cancer is the second most common cancer among women, with an estimated 123,000 new cases and 77,000 deaths in 2020 [3]. It is the leading cause of cancer mortality among women aged 15-49 years in India [4].

Screening programs aimed at the early detection and treatment of precancerous cervical lesions can effectively reduce the disease burden [3,5]. The World Health Organization (WHO) recommends screening for cervical cancer among women aged 30-49 years at least once in their lifetime using simple, affordable tests such as visual inspection with acetic acid (VIA) or the human papillomaviruses (HPV) DNA test [6]. However, screening coverage remains very low in India, with only 22% of women screened for cervical cancer nationally [5].

A lack of awareness about cervical cancer, poor knowledge regarding its prevention, sociocultural barriers, limited access to screening services, and inadequate health infrastructure and manpower have been identified as critical impediments to cervical cancer prevention and control in India [7,8]. Qualitative studies have highlighted the influence of cultural beliefs, gender norms, spousal disapproval, and reluctance to undergo gynaecological exams as factors deterring screening among women [9,10].

Understanding the interplay of knowledge, attitudes, practices, and sociocultural factors related to cervical cancer prevention is crucial for planning effective interventions. Mixed-methods studies combining quantitative instruments with qualitative, in-depth inquiry can provide valuable insights into complex health behaviors. However, there is limited evidence from mixed-methods studies on cervical cancer screening in rural, low-literacy communities in India. This study used a convergent, parallel, mixed methods design to examine the knowledge and perceptions regarding cervical cancer screening and vaccination among women in rural Gujarat. The quantitative component was used to assess the knowledge, attitudes, and practices related to cervical cancer screening, sociodemographic determinants, and risk factors for cervical cancer. The qualitative component was used to explore sociocultural factors influencing screening behaviours through in-depth interviews. Integrated analysis of quantitative and qualitative data provided a nuanced and comprehensive understanding of the multifaceted issues that shape cervical cancer prevention in marginalized rural settings. The evidence generated can inform targeted community-based interventions to promote screening in an equitable and culturally sensitive manner.

## Materials and methods

This was a community-based mixed-methods study conducted among the rural families of Gujarat from January 2023 to June 2023. The sample size was calculated using the single proportion formula (P = 34%). From the reference study, with 5% precision, the final sample size was 400 [11].

A simple random sampling technique was used to select the participants. The eligibility criteria included the following inclusion criteria: >18 years of age from rural residential areas (residents of the area for >1 year) and provided consent. 

In the qualitative arm, an exploratory qualitative study using in-depth interviews was conducted. Purposive sampling was used to identify information-rich cases for in-depth interviews. Thirty in-depth interviews were conducted with eligible women.

Tool development

A structured questionnaire to assess knowledge, attitudes, and practices related to cervical cancer screening was developed based on prior studies [12-14]. The questionnaire was subsequently translated into Gujarati and back-translated to English to verify the integrity of the questions [15]. The questionnaire is listed in Table 1. The qualitative guide was translated using the same process.

**Table 1:** Cervical Cancer Screening Knowledge, Attitudes, and Practices questionnaire

Section	Question	Response options	Answer
(A) Sociodemographic information	Age	Years	
	Marital status	Married / Unmarried / Widowed	
	Religion	Hindu / Muslim / Other (specify)	
	Occupation	Housewife / Working (specify)	
	Family type	Joint / Nuclear	
	Duration of marriage	Years	
	Education level	Illiterate / Primary / Secondary / Higher Secondary / Graduate or above	
	Socioeconomic status	Upper / Upper middle / Middle / Lower middle / Lower	
(B) Knowledge about cervical cancer	Ever heard of cervical cancer	Yes / No	
	Source of information	Health worker / Family and relatives / TV and media / Other (specify)	
	Heard about HPV vaccine	Yes / No	
	Risk factors (multiple choice)	Early sexual debut / Early pregnancy / High parity / Smoking / Oral contraceptives / Multiple partners / STIs / Family history / Lack of screening / Don't know	
	Can cervical cancer be prevented	Yes / No / Don't know	
	Prevention methods (multiple choice)	Regular screening / HPV vaccination / Using condoms / Avoiding multiple partners / Don't know	
	Is cervical cancer curable if detected early	Yes / No / Don't know	
(C) Attitudes	Cervical cancer is a serious health problem	Strongly agree / Agree / Neither / Disagree / Strongly disagree	
	Any woman can get cervical cancer	Yes / No / Don't know	
	Screening and vaccination can prevent cervical cancer	Strongly agree / Agree / Neither / Disagree / Strongly disagree	
	Willing to get HPV vaccine	Yes / No / Not sure	
	Willing to undergo screening	Yes / No / Not sure	
	Concerns about screening (multiple choice)	Fear / Embarrassment / Lack of time / Financial / Lack of family support / Fear of finding cancer / Other (specify)	
(D) Practices	Ever been screened for cervical cancer	Yes / No	
	If yes, last screening	Years ago	
	If no, reasons (multiple choice)	Lack of awareness / No symptoms / Fear / Lack of time / Financial / Lack of facilities / Other (specify)	
	Received HPV vaccine	Yes / No	
	If no, reasons (multiple choice)	Lack of awareness / High cost / Fear of side effects / Not recommended / Other (specify)	
(E) Risk factors	Age at first marriage	Years	
	Age at first pregnancy	Years	
	Number of children	Number	
	Smoking	Yes / No	
	Oral contraceptive use	Yes / No	
	History of STI	Yes / No / Don't know	
	Family history of cervical cancer	Yes / No / Don't know	
	Tobacco consumption	Yes / No	
(F) Open-ended questions	Main barriers to screening	Text	
	How to improve awareness and screening	Text	
	Role of men in prevention	Text	
	Comfort discussing with family	Text	
	Support needed for screening	Text	
	Additional comments		

Both instruments were pretested on 10% of the sample population, as recommended. Pretesting allows assessment of face and content validity, ensures comprehensibility and logical flow of questions, and determines the average time for completion [16]. Based on the pretesting, necessary modifications were made to improve the clarity of the manuscript.

The detailed procedures and training of the data collectors described previously also helped ensure the quality and reliability of the data obtained using both instruments. Appropriate data coding, entry procedures, and quantitative analysis also contribute to reliability. Achieving thematic saturation and cross-case validity analysis in qualitative research improves credibility and conformability. Data triangulation from multiple sources lends greater validity to the results [17].

Data collection

Quantitative data were collected through face-to-face interviews using a structured questionnaire. Written informed consent was obtained prior to the study. The participants were informed of the study's purpose and were assured of confidentiality. Interviews were conducted in a private setting by trained female researchers conversing in the local language. Responses were recorded on paper and later entered electronically.

Qualitative data were collected through in-depth interviews at participants' homes by trained female researchers. Oral informed consent was obtained, and interviews were audio recorded with permission. Data collection continued until thematic saturation was achieved. Field notes were maintained to document nonverbal cues and contextual observations.

Study variables

The study collected data on several key variables. Sociodemographic variables included age (categorized as ≤25 years or >25 years), marital status (married or unmarried/widowed), religion (Hindu or Muslim), occupation (housewife or working), family type (joint or nuclear), and marriage duration (<5 years, 5-10 years, or ≥10 years). The primary outcome variables were knowledge level (good or poor based on a score ≥60% on 15 knowledge questions), attitudes (positive or negative based on a score ≥60% on 10 attitude questions), screening practice (whether the participant had been screened for cervical cancer), and HPV vaccination status. Explanatory variables included whether the participant had heard about cervical cancer or the HPV vaccine, early sexual debut (first sexual encounter before age 20), early pregnancy (first pregnancy before age 20), high parity (≥3 children), low socioeconomic status (classes 3-5 on modified BG Prasad scale), education level (illiterate or literate), tobacco use, oral contraceptive use, multiple sexual partners, history of sexually transmitted infections, family history of cervical cancer, and awareness about screening. Knowledge was categorized as good if participants scored ≥60% on the knowledge questions. Attitudes were considered positive if participants scored ≥60% on the attitude questions. Screening practice was determined based on a self-reported history of undergoing cervical cancer screening tests. HPV vaccination status was also self-reported by participants.

Ethical statement

The study protocol was reviewed and approved by the Institutional Ethics Committee of Shri MP Shah Medical College (REF No. 09/01/2023). Permission was also obtained from community leaders before data collection. Written informed consent was obtained from all the quantitative study participants prior to data collection. For qualitative interviews, oral informed consent was obtained due to lower literacy levels, and this process was approved by the ethics committee.

Data handling and analysis

Quantitative Data Analysis

Quantitative data were analyzed using Epi-Data software and SPSS version 21. Descriptive statistics such as frequencies and percentages were calculated for sociodemographic variables and knowledge, attitudes, and practices regarding cervical cancer screening and vaccination.

Bivariate analysis using a chi-square test was also conducted to assess factors associated with knowledge. Variables with p<0.2 in the bivariate analysis were entered into a multivariate logistic regression model to identify independent predictors of knowledge. Adjusted odds ratios (AORs) with 95% confidence intervals (CIs) were calculated. A P-value <0.05 was considered to indicate statistical significance.

Qualitative Data Analysis

In-depth audio recordings of the interviews were transcribed verbatim and translated into English. The transcripts were read repeatedly to identify the codes and develop an initial codebook. Coding was performed manually using NVivo 12 software. Related codes were grouped into categories that further coalesced into major themes and subthemes.

Thematic analysis was performed following a combined inductive and deductive approach. An iterative process was used to identify emerging themes directly from the data (inductive) and to explore themes identified a priori from the literature review and study objectives (deductive). Comparisons were made across transcripts to ensure consistency of findings. Representative quotes were extracted to illustrate themes.

Data integration

All the data are presented in the form of tables and narratives and were used to integrate the quantitative and qualitative findings. Qualitative themes were used to contextualize and expand on the quantitative results. The triangulation test compared convergence, dissonance, and connections between quantitative and qualitative findings. Areas of divergence were further explored. The integrated results provided a nuanced understanding of the multiple factors influencing cervical cancer screening in the study population.

## Results

Table 2 shows the sociodemographic characteristics of the 400 study participants. Sixty-four percent (n = 256) were ≤25 years old. Eighty-four percent (n = 336) were married. A total of 78% (n = 310) were Hindu. A total of 89% (n = 356) were housewives. Fifty-three percent (n = 212) lived in nuclear families. Thirty-four percent (n = 136) had a marriage duration of 5-10 years.

**Table 2:** The sociodemographic characteristics of the study participants (N=400)

Variables	Category	Frequency	Percentage
Age (in years)	≤25	256	64
	>25	144	36
Marital status	Married	336	84
	Unmarried/widow	64	16
Religion	Hindu	310	78
	Muslim	90	22
Occupation	Housewife	356	89
	Working	44	11
Family type	Joint	188	47
	Nuclear	212	53
Duration of marriage (years)	<5	88	22
	5–10	136	34
	>10	176	44

Table 3 displays the knowledge, attitudes, and practices regarding cervical cancer screening and vaccination. Overall, 27% (n=108) had heard of cervical cancer. Twenty-three percent (n=92) had heard about the HPV vaccine. Of those aware of cervical cancer (n=108), 67% (n=72) received information from health workers. A total of 83% (n=332) had poor knowledge. Overall, 22% (n=88) agreed that cervical cancer was the leading cause of death. Approximately 29% (n=116) agreed that any woman could acquire cervical cancer. Forty-one percent (n=164) agreed that screening and vaccination can prevent cervical cancer. Fifty-one percent (n=204) were willing to receive a vaccination. Thirty-two percent (n=128) were willing to screen. Sixty-four percent (n=254) had a positive attitude. Nine percent (n=36) had been screened for HPV. 0% were vaccinated for HPV.

**Table 3:** Knowledge, attitudes, and practices related to cervical cancer screening and vaccination of the study participants (N=400)

Variables	Category	Frequency	Percentage
Ever heard of cervical cancer	Yes	108	27
	No	292	73
Heard about the HPV vaccine	Yes	92	23
	No	308	77
Source of the information (N=108)	Heard from a health worker	72	67
	Heard from family and relatives	28	26
	Heard from TV and media	8	7
Knowledge	Good	68	17
	Poor	332	83
Cervical cancer is the leading cause of death in women in India	Agree	88	22
	Neither agree nor disagree	264	66
	Disagree	48	12
Any woman who acquires cervical cancer	Agree	116	29
	Neither agree nor disagree	196	49
	Disagree	88	22
Screening and vaccination can prevent cervical cancer	Agree	164	41
	Neither agree nor disagree	196	49
	Disagree	40	10
Willingness	For vaccination	204	51
	For screening	128	32
Attitude	Positive	254	64
	Negative	146	36
Practice	Screened for HPV	36	9
	Vaccinated for HPV	0	

Table 4 shows the cervical cancer risk factors. Fifty percent (n=200) had an early sexual debut <20 years. Sixty-six percent (n=264) had an early first pregnancy <20 years. A total of 46% (n=184) had a parity ≥3. Fifty-three percent (n=212) had a low socioeconomic status. Sixty-one percent (n=244) were illiterate. Eighteen percent (n=72) smoked. Twenty-three percent (n=92) used oral contraceptives. Nine percent (n=36) had multiple partners. Four percent (n=16) had STIs. Six percent (n=24) had a family history of cervical cancer. One hundred percent (n=400) were unvaccinated for HPV.

**Table 4:** Risk factors for cervical cancer, including vaccination status

Risk factors	Category	Frequency (N=400)	Percentage
Early sexual debut (age at first marriage <20 years) (n=336)	Yes	200	50
	No	136	34
Early pregnancy (age at first pregnancy <20 years)	Yes	264	66
	No	136	34
High parity (number of children ≥3)	Yes	184	46
	No	216	54
Lower socioeconomic status (modified BG prasad classification)	Yes	212	53
	No	188	47
Lack of education (Illiterate)	Yes	244	61
	No	156	39
Smoking	Yes	72	18
	No	328	82
Oral contraceptive use	Yes	92	23
	No	308	77
Multiple sexual partners	Yes	36	9
	No	364	91
History of sexually transmitted infections	Yes	16	4
	No	384	96
Family history of cervical cancer	Yes	24	6
	No	376	94
Lack of awareness about screening	Yes	332	83
	No	68	17
Tobacco consumption	Yes	88	22
	No	312	78
Unvaccinated for HPV	Yes	400	100
	No	0	0

Table 5 shows the associations between risk factors and knowledge levels about cervical cancer among the study participants. Early marriage before age 20, early first pregnancy before age 20, a high parity of three or more children, lower socioeconomic status, illiteracy, smoking, no oral contraceptive usage, multiple sexual partners, history of STI, family history of cervical cancer, awareness of screening and tobacco consumption were significantly associated with poorer cervical cancer knowledge.

**Table 5:** Association between risk factors and knowledge levels of participants *P<0.05: significant, **p<0.001: highly significant, COR: crude odds ratio, AOR: adjusted odds ratio, 1: reference group.

Risk factors	Category	COR (95% CI)	AOR (95% CI)
Early sexual debut (age at marriage)	<20 years	2.5 (1.2–5.1)*	2.7 (1.3–5.6)*
	≥20 years	1	1
Early pregnancy (age at 1st pregnancy)	<20 years	3.5 (1.8–6.7)**	3.8 (1.9–7.4)**
	≥20 years	1	1
High parity (no. of children)	≥3 children	4.2 (2.1–8.4)**	4.5 (2.2–9.1)**
	<3 children	1	1
Socioeconomic status	Upper	1	1
	Lower	2.4 (1.3–4.3)*	2.6 (1.4–4.8)*
Lack of education	Illiterate	19.2 (8.5–43.4)**	21.4 (9.3–49.2)**
	Literate	1	1
Smoking	Yes	3.4 (1.7–6.9)**	3.6 (1.8–7.2)**
	No	1	1
Oral contraceptive use	Yes	1	1
	No	3.7 (1.6–8.4)**	4.1 (1.8–9.2)**
Multiple sexual partners	Yes	2.5 (0.9–6.8)	2.7 (1.0–7.4)
	No	1	1
History of sexually transmitted infections	Yes	3.9 (0.9–17.0)	4.2 (1.0–18.1)
	No	1	1
Family history of cervical cancer	Yes	1	1
	No	2.3 (0.5–10.7)	2.5 (0.5–11.9)
Awareness about screening	Yes	1	1
	No	2.5 (0.9–6.7)	2.7 (1.0–7.4)
Tobacco consumption	Yes	2.5 (1.2–5.2)*	2.7 (1.3–5.6)*
	No	1	1

Table 6 shows the associations between risk factors and cervical cancer screening practices among the participants. Early marriage before age 20, early first pregnancy before age 20, a high parity of three or more children, lower socioeconomic status, illiteracy, smoking, no oral contraceptive usage, multiple sexual partners, history of STI, family history of cervical cancer, awareness of screening and tobacco consumption were significantly associated with poorer cervical cancer screening practices.

**Table 6:** Association between risk factors and practice of the participants P<0.05*: significant, p<0.001**: highly significant, COR: crude odds ratio, AOR: adjusted odds ratio, 1: reference group.

Risk factors	Category	COR (95% CI)	AOR (95% CI)
Early sexual debut (age at marriage)	<20 years	6.3 (2.7–14.6)*	6.8 (2.9–15.8)*
	≥20 years	1	1
Early pregnancy (age at 1st pregnancy)	<20 years	4.5 (2.1–9.6)*	4.8 (2.2–10.4)*
	≥20 years	1	1
High parity (no. of children)	≥3 children	3.2 (1.4–7.4)*	3.5 (1.5–8.1)*
	<3 children	1	1
Socioeconomic status	Upper	1	1
	Lower	2.4 (1.1–5.2)*	2.6 (1.2–5.6)*
Lack of education	Illiterate	15.2 (5.1–45.6)*	16.8 (5.6–50.4)*
	Literate	1	1
Smoking	Yes	3.4 (1.2–4.8)*	3.6 (1.5–5.2)*
	No	1	1
Oral contraceptive use	Yes	1	1
	No	2.4 (1.2–4.8)*	2.6 (1.3–5.2)*
Multiple sexual partners	Yes	3.5 (1.8–5.8)	2.9 (1.6–7.4)
	No	1	1
History of sexually transmitted infections	Yes	2.9 (1.9–16.0)	3.2 (1.6–18.1)
	No	1	1
Family history of cervical cancer	Yes	1	1
	No	3.3 (1.2–10.7)	3.2 (1.5–10.9)
Awareness about screening	Yes	1	1
	No	3.5 (1.9–7.7)	4.7 (1.0–7.4)
Tobacco consumption	Yes	3.2 (1.2–5.2)*	4.4 (1.3–5.6)*
	No	1	1

Table 7 shows qualitative themes reflecting low awareness, cultural barriers, access issues, fear, and lack of spousal communication and support as key factors affecting cervical cancer knowledge and screening among rural women. Quotes highlight misconceptions, stigma, gender roles, fatalism, time constraints, language barriers, and the need for improved health education.

**Table 7:** Qualitative analysis of the participants (n=30)

Theme	Subtheme	Participant phrases
Theme 1: Low awareness and knowledge		
	Limited or no information about cervical cancer	"I do not know anything about this cancer" (P5, age 32)
		"I never heard the name cervical cancer before" (P12, age 29)
	Misconceptions	"Cervical cancer is caused by a curse or by ghosts" (P21, age 45)
		"Only women with bad character get this cancer" (P17, age 39)
Theme 2: Social and cultural barriers		
	Screening perceived as invasive	"I feel shy to go for these procedures" (P8, age 28)
	Stigma associated with screening	"People will think I have loose character if they see me going for this test" (P15, age 33)
	Lack of family support	"My husband said no need to waste money on tests" (P3, age 30)
	Preference for female doctors	"I will not allow a male doctor to do this test. I want a lady doctor" (P20, age 41)
Theme 3: Access barriers		
	Long distances to screening services	"The hospital is very far. I cannot travel that long distance alone" (P26, age 38)
	Financial constraints	"We do not have money for these expensive tests" (P18, age 36)
	Lack of female health workers	"There are no lady doctors or nurses in our village hospital" (P23, age 40)
Theme 4: Fear and fatalism		
	Cervical cancer is viewed as a "death sentence"	"If I have this cancer it is the end of my life" (P13, age 35)
	Avoidance due to fear	"I do not want to get tested because I'm scared they will find cancer" (P4, age 27)
Theme 5: Communication gap		
	Language barriers	"The doctor was speaking in English which I did not understand" (P11, age 31)
	Lack of privacy during consultations	"The doctor was talking loudly in front of others" (P9, age 29)
Theme 6: Gender roles		
	Male dominance in health decisions	"My husband has to give permission first, then only I can go for the test" (P6, age 25)
Theme 7: Prioritization		
	Focus on child and family health	"I'm too busy with housework and childcare to go for this test" (P16, age 37)
	Lack of time due to household responsibilities	"When will I get time from my work at home?" (P19, age 40)
Theme 8: Lack of self-efficacy		
	Reliance on fate/God's will about health issues	"If I am destined to get cancer, I will get it no matter what" (P22, age 43)
	Avoidance due to feeling unequipped to handle screening or treatment	"I do not know anything about these tests or cancer treatment. How can I manage all that?" (P24, age 39)
Theme 9: Lack of spousal communication		
	Hesitance to discuss reproductive health with husband	"I feel shy to talk about all this with my husband" (P27, age 41)
	Lack of spousal discussion on cervical cancer screening	"My husband does not talk to me about cancer or getting tested" (P28, age 44)
Theme 10: Lack of cervical cancer education		
	Lack of counselling by health workers	"The nurses just tell me to go for Pap test but do not explain anything" (P29, age 38)
	Need for more community outreach and awareness programs	"Th ere should be more camps and talks in our village to teach us about cervical cancer" (P30, age 42)

## Discussion

The present community-based cross-sectional study of 400 women and exploratory qualitative study of 30 women demonstrate the complex role of sociodemographic and cultural factors in determining awareness, attitudes, and practices related to cervical cancer screening among rural women in Gujarat.

Sociodemographic factors

Our results are consistent with those of previous rural studies showing low cervical cancer awareness (27%) and illiteracy (61%) among participants. Age, occupation as a housewife (89%), and prolonged child-bearing years reflected by long marriages (34% >10 years) additionally highlight the vulnerability of this group [18,19].

Awareness and knowledge

Only 27% had heard of cervical cancer, similar to the findings of Kadian et al. (25%). Sixty-seven percent of the participants received information from health workers, mirroring the findings of Chawla et al., where ASHAs were the leading source. This finding points to missed educational opportunities during existing health contacts [20,21].

The 83% with poor knowledge corroborates previous studies: a recent quantitative study in the UAE revealed that 80% of surveyed women were unaware of a ‘precancerous cervical lesion’ [22]. Similarly, studies in Gulf countries have shown limited knowledge about cervical screening among women [23-25]. For instance, the research by Jassim et al. found that 64% of Bahraini women had never heard of the term ‘pap smear test’ [25]. The findings of these studies emphasize the critical need to address the knowledge gap regarding cervical cancer in public health (65-75%).

Attitudes and beliefs

The finding that only 64% of respondents in our study had positive attitudes toward cervical cancer screening, along with prevalent misconceptions about contracting the disease, reflects the stigma, fear, and cultural barriers that persist across rural communities. This is consistent with previous research identifying similar challenges in LMICs. A systematic analysis of global adherence to cervical cancer screening in 2019 estimated that only 24.91% of eligible women in LMICs participated in screening, compared to 75.66% in high-income countries. Despite adequate knowledge about signs, symptoms, and risk factors among health professionals in India, the actual practice of screening remains low, at 12.70%, largely due to social stigma and other barriers. These findings align with our study's results, underscoring the need to address not only knowledge gaps but also the cultural and infrastructural challenges that hinder screening uptake. A community-based study among tribal women in coastal Karnataka, India, further corroborates our findings, revealing suboptimal knowledge of cervical cancer, favourable attitudes toward screening, but low actual screening practice (12.70%). Our study contributes to the growing body of evidence highlighting the complex interplay of factors influencing cervical cancer screening in rural and underserved communities, emphasizing the importance of targeted interventions that consider local contexts and barriers [25-29].

Practices

The screening rate of 9% found in our study is consistent with the low rates reported in previous research, particularly in low and middle-income countries (LMICs) and rural communities. A systematic analysis of global adherence to cervical cancer screening in 2019 estimated that only 24.91% of eligible women in LMICs participated in screening, compared to 75.66% in high-income countries. The African region had the lowest adherence at 5.28%, highlighting the significant disparities in screening uptake across regions.

Our findings also align with studies conducted among health professionals and tribal women in India. Despite adequate knowledge about cervical cancer signs, symptoms, and risk factors among health professionals (75.15%), the actual practice of screening remains low at 12.70%. This discrepancy between knowledge and practice underscores the influence of social stigma and other barriers on screening uptake. A community-based study among tribal women in coastal Karnataka further corroborates these findings, revealing suboptimal knowledge of cervical cancer, favourable attitudes toward screening, but low actual screening practice (12.70%) [26-29].

The low screening rate in our study reflects the complex interplay of factors hindering cervical cancer screening in rural communities. Lack of awareness, cultural factors, and infrastructure challenges have been identified as significant barriers to large-scale screening programs in India. Our findings emphasize the urgent need for targeted interventions that address these challenges and empower women to overcome the stigma and fear associated with cervical cancer screening.

Our study contributes to the growing evidence highlighting the need for comprehensive strategies focussing on awareness campaigns, infrastructure improvement, and empowering women to overcome these challenges. By addressing these barriers, we can work towards improving cervical cancer screening uptake and ultimately reducing the burden of this preventable disease in underserved communities.

Qualitative insights

The qualitative findings of our study highlight several psychosocial barriers to cervical cancer screening that are consistent with previous research. Misconceptions about cervical cancer, its causes, and prevention were identified as significant barriers to screening participation. These misunderstandings can lead to a lack of perceived risk and a belief that screening is unnecessary, ultimately hindering uptake. The social stigma associated with cervical cancer was another prominent barrier, discouraging open discussions about the disease and screening. Women often expressed fear of being stigmatized or ostracized by their communities if they were to undergo screening or receive a positive diagnosis.

Traditional gender roles and norms were found to influence health-seeking behaviour among women in our study. Many participants expressed that their male partners played a crucial role in decision-making related to healthcare, including cervical cancer screening. This finding aligns with the research conducted by Dsouza et al., which emphasized the importance of male engagement in increasing open dialogue and health literacy related to HPV and cancer screening. Effective communication between spouses was identified as a vital factor in encouraging screening uptake, highlighting the need for interventions that target both women and their male partners [30].

Our study also revealed that family support and approval significantly influenced women's health-seeking behaviour, consistent with the findings of Dsouza et al. [30]. Women who received encouragement and support from their families were more likely to participate in screening, while those who faced opposition or lack of understanding from family members were less likely to seek preventive care. This underscores the importance of engaging family members, including male partners, in awareness campaigns and educational initiatives to enhance screening participation.

The qualitative findings of our study suggest that effective interventions should address the complex interplay of psychosocial factors influencing cervical cancer screening uptake. Health behavioural models, such as the Theory of Planned Behaviour and Health Belief Model, can inform targeted interventions that address women's attitudes, perceived benefits, emotions, and context-specific barriers. Community engagement strategies that involve family members, male partners, and influential community leaders can help to challenge misconceptions, reduce stigma, and promote a supportive environment for screening.

In summary, our qualitative findings highlight the significant psychosocial barriers to cervical cancer screening, including misconceptions, stigma, traditional gender roles, and family influence. These findings are consistent with previous research and emphasize the need for comprehensive interventions that address these barriers at multiple levels. By engaging communities, challenging social norms, and promoting open dialogue, we can work towards increasing cervical cancer screening uptake and ultimately reducing the burden of this preventable disease in underserved populations.

Limitations

This study, while providing valuable insights, has several limitations that should be considered when interpreting the results. As a cross-sectional study, it establishes associations between variables but cannot determine causation or temporal relationships. The reliance on self-reported data for cervical cancer screening may have led to an overestimation of actual screening rates due to social desirability bias, although efforts were made to validate these reports using medical records. The study's generalizability is limited by its focus on one district, potentially not capturing the full diversity of rural experiences across Gujarat or India. However, the qualitative findings offer better transferability to similar contexts. The validated knowledge questionnaire, while useful, may not have fully captured community-specific beliefs and cultural nuances that influence screening behaviors. An important limitation is the absence of male perspectives, despite spousal communication emerging as a significant theme. Including male views would have provided a more comprehensive understanding of the sociocultural environment affecting women's health-seeking behaviors. Lastly, self-selection bias cannot be ruled out, as study participants may have been more aware of cervical cancer issues than non-participants, potentially missing the perspectives of hard-to-reach women who may face different or more severe barriers to screening.

Recommendations

Based on the study findings and limitations, several recommendations can be made to advance understanding and improve cervical cancer prevention efforts in rural India. Longitudinal cohort studies of rural women are needed to provide more robust evidence on temporal predictors of screening intention and behaviour, addressing the limitations of cross-sectional data. To gain a more representative picture of cervical cancer awareness and screening coverage, larger statewide surveys should be conducted. These would help identify regional variations and inform targeted interventions. The development of locally validated knowledge assessment tools and ethnographic studies is recommended to better elucidate cultural barriers and drivers specific to different contexts within rural India. Given the importance of spousal communication highlighted in this study, diadic research on couple communication patterns could inform the development of family-centred interventions for cervical cancer prevention. To address access barriers, piloting outreach camps, mobile clinics, and integrating screening with existing public health programs could expand screening access. These recommendations, if implemented, could significantly enhance cervical cancer prevention efforts, leading to more effective and culturally appropriate interventions in rural India.

## Conclusions

The present mixed-methods study provides comprehensive insights into the complexity of individual and interpersonal factors affecting cervical cancer prevention among rural women. Sociodemographic vulnerability, poor knowledge, and cultural misconceptions intersect to deter early screening. Awareness creation must occur respectfully by addressing barriers such as stigma, fear, and gender roles impeding access to prevention services. Long-term strategies require going beyond educating women to promote male engagement, informed spousal communication, and family support for women's health-seeking. Integrating services within trusted community platforms along with increased budgetary allocation can drive the policy and programmatic efforts needed to extend the benefits of cervical cancer prevention to marginalized groups in rural India. The insights from this study can inform the development of socioculturally resonant information campaigns and grassroots interventions that respond to the worldviews and lived realities of such underserved women.
